# Clinical Application of Bone Marrow Mesenchymal Stem/Stromal Cells to Repair Skeletal Tissue

**DOI:** 10.3390/ijms21249759

**Published:** 2020-12-21

**Authors:** Agnieszka Arthur, Stan Gronthos

**Affiliations:** 1 Mesenchymal Stem Cell Laboratory, Adelaide Medical School, Faculty of Health and Medical Sciences, University of Adelaide, Adelaide, SA 5001, Australia; agnes.artrhur@adelaide.edu.au; 2 Precision Medicine Theme, South Australian Health and Medical Research Institute, Adelaide, SA 5001, Australia

**Keywords:** bone marrow mesenchymal stem cells, bone marrow microenvironment, skeletal tissue regeneration, tissue engineering, scaffolds, biomaterials

## Abstract

There has been an escalation in reports over the last decade examining the efficacy of bone marrow derived mesenchymal stem/stromal cells (BMSC) in bone tissue engineering and regenerative medicine-based applications. The multipotent differentiation potential, myelosupportive capacity, anti-inflammatory and immune-modulatory properties of BMSC underpins their versatile nature as therapeutic agents. This review addresses the current limitations and challenges of exogenous autologous and allogeneic BMSC based regenerative skeletal therapies in combination with bioactive molecules, cellular derivatives, genetic manipulation, biocompatible hydrogels, solid and composite scaffolds. The review highlights the current approaches and recent developments in utilizing endogenous BMSC activation or exogenous BMSC for the repair of long bone and vertebrae fractures due to osteoporosis or trauma. Current advances employing BMSC based therapies for bone regeneration of craniofacial defects is also discussed. Moreover, this review discusses the latest developments utilizing BMSC therapies in the preclinical and clinical settings, including the treatment of bone related diseases such as Osteogenesis Imperfecta.

## 1. Therapeutic Potential of Bone Marrow Mesenchymal Stem Cells

Bone mineral is composed of inorganic (hydroxyapatite crystals) and organic components (predominantly a collagen type 1 dependent extracellular matrix). The microenvironment within the bone is also complex, consisting of multiple cell types. These include the stromal lineage, of osteogenic (osteogenic progenitors, osteoblasts, bone lining cells and osteocytes), adipogenic and chondrogenic derivatives, and the hematopoietic lineage of the erythroid, myeloid and lymphoid derivatives. The microenvironment also consists of endothelial, perivascular, and neural populations that collectively maintain skeletal integrity and assist in skeletal repair following injury. These cell types interact with bone marrow mesenchymal stem/stromal cells (BMSC) to maintain mechanical strength and skeletal integrity by continuously remodeling the skeleton throughout life [1,2,3,4,5,6]. The physiological process of primary bone healing, where no callus is formed, consists of the initial inflammatory phase, followed by the infiltration of monocytic derived pre-osteoclasts to the injury site. The pre-osteoclasts mature forming multinucleated osteoclasts resorbing the bone matrix. This process is followed by the reversal phase where BMSC and osteoprogenitors are sequestered, localize, integrate and undergo osteogenic differentiation, synthesizing bone matrix (osteoid) which is subsequently mineralized [7]. Secondary bone healing following fracture requires distinct highly coordinated yet overlapping physiological process. The repair begins with the inflammatory phase, where necrotic tissue is removed and angiogenesis is initiated. This is followed by the infiltration of mesenchymal stem cells (MSC)/progenitors that facilitate endochondral ossification, stabilizing the fracture site through the formation of a calcified cartilage matrix. This soft callus is subsequently resorbed by chondroclasts, allowing for the formation of a hard callus; a woven mineralized matrix synthesized by osteoblasts. The subsequent remodeling phase utilizes osteoclasts to resorb the immature woven bone slowly replacing it with osteoblasts derived lamellar bone [8,9,10]. These processes of bone healing are spatially and temporally regulated and rely on numerous cellular and molecular interaction [10,11]. 

In particular circumstances this bone healing process is impaired and requires assistance, this includes but is not limited to non-union fractures or critical sized bone defects, infection, musculoskeletal diseases such as osteoporosis; osteosarcomas, osteomyelitis, congenital disorders such as osteopetrosis, osteogenesis imperfecta, cleft lip or palate, in addition to rheumatoid arthritis and osteoarthritis. The clear medical need to assist in this skeletal repair has underpinned the development of novel approaches and refinement of existing approaches to improve musculoskeletal tissue engineering strategies.

BMSC display many favorable characteristics for regenerative therapy, which have been wildly described including their multipotency, anti-inflammatory and immune-modulatory properties [12,13]. This stem cell population also has the ability to support hematopoiesis and stimulate angiogenesis. Furthermore, the release of paracrine factors by BMSC influences the surrounding microenvironment, which is a characteristic of particular interest for organ repair [14]. It has been shown that while BMSC may not engraft with high efficiency following transplantation, they can support the survival of the surrounding tissue through the release of these paracrine factors [15,16]. Due to these therapeutically desirable properties, BMSC have been utilized and investigated in the treatment of a range of diseases including cardiac, lung, neural, hematopoietic, graft-versus-host disease, in addition to tendon, ligament and musculoskeletal tissue repair [17].

In 2006 The International Society for Cellular Therapy defined human derived BMSC to consist of the following criteria (1) isolated cells are plastic adherent in culture, (2) these cells express cluster of differentiation (CD) 73 CD73, CD90, and CD105 in greater than 95% of the cell population, (3) greater than 95% of the cells lack the expression of CD14 or CD11b, CD79a or CD19, CD34, CD45, and Human leukocyte antigen (HLA)-DR, and (4) the cultured BMSC have the ability to differentiate into osteoblasts, adipocytes and chondroblasts [18]. These criteria, while important are limited and lack indices for stemness. A number of additional cell surface markers have since been identified which isolate clonogenic BMSC that are able to self-renew, support hematopoiesis and possess multi-lineage differentiation potential. These include STRO-1, CD146, CD106, platelet-derived growth factor receptor (PDGF-R), epidermal growth factor receptor (EGF-R), insulin-like growth factor receptor (IGF-R), CD49a/CD29, nerve growth factor receptor (NGF-R), CD271, CD44, [19,20,21,22,23,24,25,26,27]. In addition to their capacity to form bone, cartilage and adipose tissue, BMSC have been shown to differentiate into tendon, myogenic and neural cells in vitro and in some circumstances in vivo in response to the surrounding environmental factors [14]. While BMSC were initially isolated from the bone marrow, MSC-like populations have subsequently been identified in other tissues including adipose tissue, dental pulp, referred to as dental pulp stem cells (DPSC), periodontal ligament, perivasculature, umbilical cord, placenta, synovial membrane [21,25,28,29,30,31,32]. These MSC-like populations play an important role in tissue engineering and regenerative therapy, however the present review will focus predominantly on BMSC unless otherwise stated.

## 2. Skeletal Tissue Regeneration—Advancements over the Last Decade

Over the past decade a greater understanding has emerged of the capabilities of BMSC in skeletal regeneration with mainly pre-clinical studies and a handful of clinical studies underway, addressing the potential of using BMSC therapy in conjunction with ceramic, biodegradable, synthetic and or matrix scaffolds for the treatment of musculoskeletal tissue repair [3]). The ever expanding development of BMSC based therapies, for the treatment and repair of musculoskeletal tissue is evidenced by numerous human clinical studies addressing different bone regeneration applications (Table 1 and Table 2), as well as animal studies seeking to improve veterinary practice [33,34].

Whilst there are challenges associated with the generation of diverse tissue engineering strategies, considerable advances have been made to repair and regenerate skeletal tissue using numerous approaches, which are continuing to be developed and improved with particular attention given to elucidating the mechanisms by which regeneration is facilitated [35,36,37,38]. These include choosing the right source of the stem cell, whether autologous or allogeneic; how these stem cells are localized to the injury site directly or indirectly through migration; and whether endogenous BMSC are recruited to participate in the regeneration process [39,40]. The timing of administrating exogenous BMSC is also critical with respect to the hematoma and inflammatory response to achieve the greatest bone repair [5,41]. Importantly, efforts are being made to determine how BMSC are subsequently integrated to form the correct cell configuration and able to differentiate, repair and recapitulate the functional skeletal tissue and how mechanical loading is exerted upon the MSC engineered bone [42]. Another consideration is to improve/restore or modulate the diseased or disrupted microenvironment prior to the commencement of the regenerative therapy, to ensure greater efficacy in skeletal repair [43]. Furthermore, a permissive vascular environment is imperative for bone formation, where vascular supply assists in bone regeneration by mitigating hypoxic conditions and necrosis within the scaffold, in addition to the strong coupling between angiogenesis and osteogenesis. The structure of the cortical bone, the trabecular bone and the marrow space differ from one another, yet all need to be regenerated following non-union fracture. Numerous cell types also need to be supported within these diverse structures. For this to occur the distinctive and specific fabrication of biomaterials and delivery methods are required to recruit endogenous BMSC or deliver exogenous BMSC. 

### Delivery Modes of Bioactive Signals and/or BMSC

**Figure 1:**
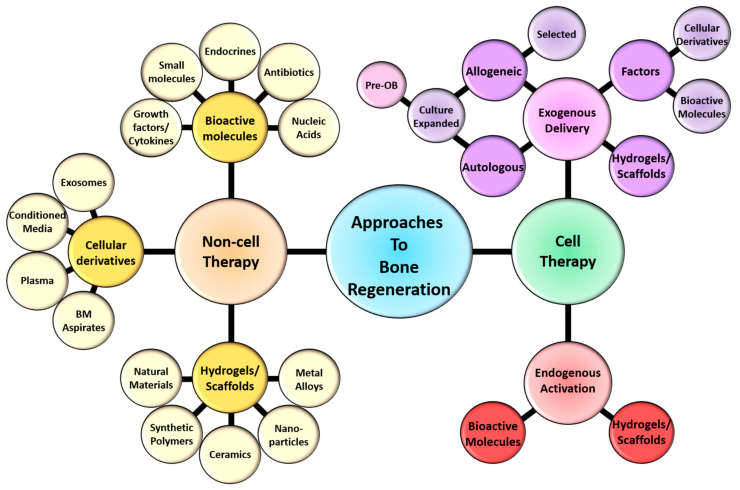
Schematic representation of the current approaches to bone regeneration. Non-cell therapy approaches to bone regeneration encompass bioactive molecules cellular derivatives and hydrogels/scaffolds/implants that can interact with and regulate/influence cellular responses. Cell therapy approach comprise the delivery of exogenous cells, either autologous or allogeneic in combination with non-cell therapy approaches. Alternatively the activation of endogenous cells (stem cells, endothelial cells, hematopoietic populations and/or mesenchymal populations) through non-cell therapy approaches have been and are currently being developed to regenerate the appropriate skeletal tissue. The bioactive molecules include a range of growth factors/cytokines, small molecules/drugs, endocrines/hormones, antibiotics and nucleic acid/genetic manipulation. The cellular derivatives incorporate components that have been derived from cells within the bone, these include exosomes, conditioned media, plasma/platelet rich plasma (PRP) and aspirates from the bone marrow (BM). The hydrogels/scaffolds/implants embodies the fabrication of injectable, microbeads, nanogels, fibers, biofilms, membranes, sponges, bone grafts and solid scaffolds that are derived from either natural materials, synthetic polymers, ceramics, nanoparticles, metal alloys or composites of these components. These bio-compatible hydrogels/scaffolds are also manipulated to incorporate and deliver cells, cellular derivatives and/or bioactive molecules in a temporal and spatial manner to enrich bone regeneration.

The methods developed to recruit endogenous BMSC and deliver exogenous BMSC systemically or locally (Figure 1) include cell-free strategies, magnetic cell labeling and tissue specific targeting, aptamer-nanoparticles, small bioactive molecules, injectable agents, the use of platelet-rich plasma (PRP) or bone marrow aspirates, BMSC secreted exosomes, and bio-engineered scaffold approaches, including three dimensional (3D) bioprinting (bioinks) [43,44,45,46,47,48,49,50,51,52,53,54,55,56,57,58]. 

The importance of incorporating/utilizing bioactive signals for enhanced bone regeneration has expanded considerably in recent times. These bioactive signals include growth factors, small molecules, endocrines, antibiotics and nucleic acids. The strategies developed thus far to induce endogenous BMSC infiltration and skeletal repair through the delivery of these bioactive signals include an array of diverse approaches reviewed by Dang and colleagues [55]. These include surface presentation of the bioactive signal, or preprogrammed controlled and sustained release of the bioactive signals via responsive release, due to endogenous signals or external stimuli such as temperature, pH, ultrasound, electric or magnetic field, light irradiation or biomolecules. Other developments involve gene delivery strategies, facilitated through gene transduction or transfection utilizing viral or non-viral vectors, respectively, to regulate molecular expression and cellular function, such as proliferation and osteogenic differentiation, which promote skeletal repair. 

Hydrogels and scaffolds use a range of natural and synthetic materials and biopolymers to achieve bone regeneration [56,57]. Natural materials include proteins, such as collagen, gelatin, laminin, keratin, elastin, fibroin, fibrin, heparin; or polysaccharides such as hyaluronan, chitosan and alginate, while those with microbial activity including cellulose, gellan gum and dextran [58,59,60,61,62,63]. Synthetic biopolymers include poly(ethylene glycol) (PEG), polyacrylamide (PAM), plyvinyl alcohol (PVA), poly lactic acid to name a few [57,58,64,65]. Furthermore, minerals such as calcium (Ca), phosphorus (P), magnesium (Mg), potassium (K), zinc (Zn) and copper (Cu) are important in bone structure. Ceramics with structural similarity to these minerals, such as hydroxyapatite (HA), calcium phosphate (CaP), tri-calcium phosphate (TCP) have been sourced for bone regeneration [66]. However, there are distinct differences in the osteogenic promoting properties of these materials in vivo between species, which often lead to encouraging pre-clinical studies but poor human clinical outcomes [67,68]. Other ceramics including coral, bioactive glass ceramics, silicon dioxide (SiO_2_), zirconium oxide (ZrO_2_), titanium dioxide (TiO_2_) and metal alloys, such as titanium (Ti) and Mg have also been utilized in scaffold synthesis [63,64,65,69]. 

Hydrogels and scaffolds possess desirable qualities to either assist in the regeneration of bone or to provide a bone substitute [58,65,70]. Hydrogels are versatile in geometry and can be used as an injectable or for transplantation. They provide the necessary moisture required to mimic the tissue-like extracellular matrix microenvironment, while solid porous scaffolds attempt to mimic bone. Both allow for cellular induction, dynamic multi-cellular interactions, which can then lead to cellular differentiation in situ. However it has become apparent in recent years that fabrication, biocompatibility, bio-degradability and bio-integration, immunogenicity, cytotoxicity, gelation time, porosity, incorporation of metal ions, payload release profile, cellular infiltration, delivery of a vascular permissive environment, bone adhesiveness, degradation time, mechanical and anti-bacterial properties need to be considered when developing hydrogels, scaffolds or composites [70,71,72,73,74,75,76]. The natural and synthetic materials are fabricated into a range of structures including but not limited to injectable hydrogels, microbeads, nanogels, hydrogel fibers, biofilms, membranes, solid porous scaffolds or sponges. These scaffolds are prepared by microfluidics, in situ polymerization, electrostatic droplet extrusion, emulsification and coaxial air jetting, physical and chemical crosslinking, electrospinning, solvent casting and particulate leaching, gas-foaming, powder compaction, emulsion freeze-drying, thermal phase separation, laser sintering, stereolitography, and 3D printing [56,59,60]. 

New tissue engineering approaches are being employed to generate composite hydrogels/scaffolds combining biopolymers, materials, small molecules or cells for enriched skeletal regeneration [55,65,67]. For example, Liu and colleagues have modified the chitosan (CS) hydrogels, incorporating catechol (CA), to improve the adhesive properties of the hydrogel, and zeolitic imidazolate framework-8 nanoparticle (ZIF-8 NP), where zinc displays antibacterial properties, and contributes to angiogenesis and osteogenesis [77]. Supportive in vitro data using rat BMSC and an in vivo studies using a rat calvarial defect model demonstrated that the CA/CS hydrogel modified by ZIF-8 NP at a medium (1.2 mg) composition (CA-CS/ZM) hydrogel combined with bone graft was more stable, displayed neovascularization and osteogenesis, and enhanced bone regeneration [77]. Furthermore elastin-like proteins (ELP) and surfactants fabricating structured organofibers have recently been developed. These organofibers are strong, elastic and can be programed with molecular and protein engineering approaches and survival of BMSC. Although still at a proof-of-concept stage, this tissue engineering strategy holds great promise [78]. 

Studies utilizing silk fibroin, have shown promising results where this material appears to be as efficient in assisting in bone formation as commercially available collagen membranes [79]. Silk fibroins in combination with HA nanocomposite particles can be adjusted to facilitate the formation of different bone types or required regeneration period [80]. Alternatively silk fibroins have been manipulated in vitro to form biomaterial rolls resembling the appearance of osteons, which enabled not only osteogenesis of human MSC (hMSC) but also the survival and directional growth of neurite processes [81]. Other examples include the development of bioglass functionalized gelatin nanofibrous scaffolds, which promoted ectopic bone formation in rats [64], and the use of the BMSC derived extracellular matrix in combination with a 3D-printed HA scaffold to promote strong osteogenic ability and appropriate “tissue-space” structure [82]. Furthermore, researchers have also suggested bone synthesis can be improved via a biphasic dual delivery scaffold systems [83,84]. More specifically one approach used a system containing two scaffolds, one consisted of a Collagen type I hydrogel that was overlaid onto the surface of the other beta-TCP (β-TCP) scaffold. The β-TCP scaffold contained a slow release of osteogenic peptide (functionally synthesized equivalent of bone morphogenetic protein-2 (BMP-2)), while the hydrogel was loaded with a quick release angiogenic peptide (functionally synthesized equivalent of vascular endothelial growth factor (VEGF)), thus appropriately influencing both osteogenesis and angiogenesis, respectively [84]. In the preclinical setting others are investigating the multifactorial approach of utilizing hMSC in conjunction with endothelial progenitor cells cultured within a macroporous scaffold under “dynamic conditions” in a biaxial bioreactor prior to sub-cutaneous transplantation in immunocompromised mice. This study demonstrated enhancing vascularization improves bone formation both in vitro and in vivo [73]. The importance of the vasculature for bone regeneration is also supported by other studies. Where periosteum derived cells, albeit from mouse, undergo osteogenesis, these cells also contribute to various facets of vascularization. The production of VEGF promoted angiogenesis by adopting pericyte characteristics which support the vasculature [85]. 

Alternative approaches have provided an environment that is less likely to cause infection such as osteomyelitis. This was addressed by modifying nanoscale HA with sliver, which is known to have antimicrobial properties, and combining with an electrospun scaffolds. As a proof-of-concept study this scaffold was shown to be non-toxic to rat BMSC and improved osteogenic differentiation capacity of cultured BMSC, while significantly reducing bacterial populations [86]. Using a similar concept, ZnO/nanocarbon modified fibrous scaffolds have demonstrated osteogenic and antibacterial properties, albeit in vitro [87]. While there are still limitations with regard to the functional capacity of hydrogels and scaffolds, the unique and versatile configurations and continuous refinement in combination with BMSC treatment holds considerable promise for bone regeneration [70,74].

## 3. BMSC Treatment for Bone Related Skeletal Diseases/Disorders/Trauma

### 3.1. Repair of the Long Bones and Vertebrae

A number of musculoskeletal conditions such as osteoporosis, osteomyelitis, osteosarcoma, diabetic fracture and congenital disorders such as osteogenesis imperfecta result in a compromise or delay in skeletal formation/repair [88,89,90,91]. While a majority of reports have utilized exogenous autologous or allogeneic MSC, other studies have attempted to recruit endogenous MSC through the administration of bioactive signals, including but not limited to BMP, parathyroid hormone (PTH), C-X-C Motif Chemokine Ligand 12 (CXCL12), VEGF, PDGF-Rβ, Interleukin 10 (IL-10), and Leucine Rich Repeat Containing G Protein-Coupled Receptor 5 (Lgr5) signaling pathways, mediated by the above-mentioned biomimetic delivery methods [17,92,93,94,95]. Several of these bioactive signals and delivery approaches have been highlighted in this review.

Preclinical studies and clinical trials have demonstrated that BMPs, particularly BMP2 and BMP7, improve outcomes of sinus lifts, spinal fusion and open tibial fractures [96,97,98,99,100,101]. However there have been side-effects with these treatments including heterotropic ossification and ectopic bone formation, as well as bone resorption and spinal swelling [102,103,104]. Another family member, BMP6 (OSTEOGROW) coupled with a biocompatible autologous peripheral blood coagulum derived carrier has been administered to a critical size bone defect to augment bone repair in a preclinical rabbit model through the stimulation of endogenous MSC differentiation without initiating inflammation nor resorption [105]. This product is currently undergoing clinical assessment (EudraCT number: 2017-000860-14) to investigate the safety, tolerability and efficacy of a single administration of OSTEOGROW in conjunction with lumbar fusion to treat degenerative disc disease. 

A novel and alternative approach of stimulating endogenous MSC with BMPs is via local targeted gene therapy utilizing a microbubble-enhanced, ultrasound (Sonoporation) mediated gene delivery of *bmp6* and a collagen scaffold that was inserted within the non-union fracture site of a large animal model (miniature pigs). Bez and colleagues demonstrated that *bmp6* gene delivery to the fracture site 2-weeks post fracture predominantly targeted the MSC. This approach appeared to be safe, feasible and effective at improving fracture healing, resulting in bone union comparable to the gold standard autograft treatment [106]. Interestingly, genetically modifying allogeneic porcine MSC to over express *bmp6* in a preclinical porcine model mimicking vertebrae compression fractures, as experienced by osteoporotic patients, resulted in greater vertebral bone repair 6 months post transplantation [107]. However, it’s worthwhile noting that these observations were based on comparison to the fibrin gel only control rather than fibrin-MSC, therefore the contribution of *bmp6* or MSC is unclear in this pilot study [107]. Furthermore, an alternative paracrine approach suggests that exosomes secreted from BMSC are thought to improve osteogenesis and angiogenesis during skeletal repair of nonunion fractures which has been attributed in part to the BMP-2 and VEGF signaling pathways [108]. However, limitations of this approach involve the accumulation of exosomes within the liver and lungs. Therefore building on this concept researchers have combined aptamer complexes specifically targeting the bone with BMSC derived exosomes to improve skeletal outcomes in an osteoporotic mouse model [109]. 

Similar lines of investigation, have employed combined release of an osteoinductive agent, BMP2, and chemoattractant of MSC, CXCL12 [95,110], via a newly engineered chitosan oligosaccharide/heparin (CSO/H) nanoparticle-modified chitosan-agarose-gelatin (CAG) scaffold. These studies resulted in the migration of allogeneic BMSC towards the transplanted scaffold mediated through CXCL12 signaling, while BMP2 signaling promoted osteogenesis within the scaffold in vitro [111]. Furthermore, the administration of a MSC membrane sheet combined with injection of CXCL12 synergistically enhances bone repair when compared to monotherapy in a nonunion fracture rat model [112]. These findings are in agreement with observations where CXCL12 release from an alginate hydrogel within the nonunion fracture results in the recruitment of inflammatory cells and endogenous MSC, which were able to retain their multipotent state [113]. Thus the co-administration of BMP2 and MSC would be appropriate to instigate osteogenic differentiation of the recruited or administered BMSC. CXCL12 has also been reported to promote early vascular formation when co-administered with MSC, which was equivalent to MSC co-administration of endothelial progenitor cells [114]. In contrast, the Food and Drug Administration (FDA) approved C-X-C chemokine receptor type 4 (CXCR4) antagonist, AMD3100, also activates MSC mobilization within the circulation, which assisted in femoral and spinal bone regeneration [115,116]. These observations suggest that CXCL12 can be utilized in multiple ways to help facilitate MSC mediated bone regeneration.

The first anabolic treatment for osteoporosis was the administration of PTH, more specifically the N-terminal 1–34 fragment commonly known as Teriparatide [117,118]. While PTH is well known for its catabolic action, the anabolic action of PTH is mediated by PTH acting through its receptor Parathyroid Hormone 1 Receptor (PTH1R) expressed by MSC and osteogenic lineages, to promote proliferation and osteogenic differentiation [119]. A meta-analysis study based on eight randomized trials has shown that injection of PTH is also safe and effective in treating fracture repair. Patients treated with PTH displayed accelerated fracture healing [120]. Other researchers are currently investigating whether local delivery/release of PTH via either cell-free biomimetic nanofibrous scaffold or hydrogel embedded in a porous poly scaffold are more advantageous for bone healing compared to systemic administration via injection of PTH using rodent preclinical models [121,122]. While PTH has been used to stimulate endogenous MSC function, PTH has recently been combined with MSC therapy, where the co-administration of MSC and PTH significantly enhanced bone repair when compared to the monotherapies or no treatment in a preclinical rat and minipig lumber vertebral defect model [123]. The authors proposed that the less invasive nature of systemic administration via a combinatorial therapy could be a better approach to treating fragility fractures, particularly in the elderly. This notion is also supported by reports of enhanced bone regeneration in a rat non-union rib defect model following systemic administration of hMSC (IV injections) in combination with PTH treatment (subcutaneous injections). Furthermore, these repaired ribs exhibited stiffer tension than non-fractured ribs during compression and bending simulations [124]. Whilst the MSC and PTH treatments were delivered separately and by alterative means, this combinational approach was found to be substantially more beneficial than either treatment alone.

Alternative strategies have attempted to mimic the bone remodeling process, where bone anabolic osteoblast function and catabolic osteoclast function are reproduced. One report utilized mouse derived BMSC transplanted at a 10:1 ratio with mouse bone marrow mononuclear cells (BMMNC), containing pre-osteoclasts, combined with a decalcified bone matrix scaffold, which resulted in greater bone formation in a rat critical size defect model over BMSC transplanted alone or with no BMSC [125]. Supportive proteomic analysis and in vitro studies utilizing neutralizing antibodies suggest that the mechanism by which the combination of BMSC and pre-osteoclasts facilitates greater bone repair was mediated by the pre-osteoclast secretion of CXCL12, which promoted MSC migration; and Insulin Like Growth Factor Binding Protein 5 (IGFBP5), which enhanced MSC osteogenic differentiation [125]. While others have also demonstrated CXCL12 stimulates MSC migration [95,110], CXCL12 is also abundantly expressed by MSC and osteogenic populations [126,127] and is known to attract monocytes and promote osteoclast development and function [128,129]. Therefore CXCL12 may have multiple roles that should be considered [130]. In another study, a cell free strategy using a novel medullary needle implant composed of degradable Mg and the bisphosphonate, zoledronic acid (ZA) augmented fracture healing, bone quality and mechanical strength of fracture femur of osteoporotic rats. In this model the dual biological functions of the Mg stimulated osteogenesis, while locally delivered ZA inhibited osteoclast resorption. This proof-of-concept study may provide alterative avenues for orthopedic implants [131]. Interestingly, both studies influenced osteoclast function in opposing ways, while still demonstrating enhanced bone regeneration. These observations suggest that the factors released by the osteoclastic population improve osteogenic outcomes, which are associated with the reversal stage of skeletal repair. Certainly it has been reported that co-culture of BMSC with either bone marrow, platelet rich plasma or fibrin improves skeletal outcomes in fracture healing, in preclinical and clinical settings [132,133,134,135]. In the clinical setting, the use of autologous BMMNC coupled with allogenic bone grafts in a phase I/II clinical trial, showed bone regeneration, lack of pseudoarthrosis and reoccurring pain in treated patients [38]. Notably the BMMNC comprise a heterogeneous cellular population largely comprised of mature monocytic and lymphoid populations with minor subsets of stem cells and progenitors of endothelial, mesenchymal and hematopoietic origin. The BMMNC or PRP produce pro-angiogenic and -osteogenic factors that support bone synthesis [38,135]. However, a systematic review of the administration of bone marrow aspirates for skeletal defects or nonunion fractures supports the proposal of MSC within the bone marrow aspirate contribute to skeletal repair. Although they do appreciate that the biological events and subsequent therapeutic outcomes requires further investigation [136].

Various studies have suggested the importance of culturing BMSC prior to in vivo transplantation. Preclinical nonunion rat model, subperiosteal osteotomy and critical size femoral defect rabbit models have been used to show that this is also an efficacious approach to repair skeletal tissue. While pre-differentiating BMSC towards osteogenic lineage in combination with biomaterials is one approach to facilitate skeletal repair clinically [137,138,139], the maturation of BMSC in vitro diminishes their capacity to survive when transplanted in vivo compared to immature BMSC. The paracrine effects of BMSC are also critical in expediting neovascularization within the scaffold [139]. These observations are supported by other studies investigating skeletal repair through intravenous injection of allogeneic osteogenic-induced BMSC in a preclinical rat model of osteoporosis [140]. Furthermore, an ovine study using STRO-4 selected ovine BMSC in combination with bone-derived extracellular matrix (ECM) hydrogels for 3 months demonstrated comparable bone repair to that of the gold-standard autografts in a tibial segmental defect model. However, the limitations of this study were the deficiency in functional blood supply and potentially the STRO-4 selected cells displaying a chondrogenic phenotype in vitro, which may have delayed the repair process [141]. Alternatively the use of bone-derived ECM hydrogels may have only promoted a suboptimal bone regeneration response, where the use of prospectively isolated allogeneic ovine MSC seeded onto HA/TCP collagen scaffolds yielded significant bone regeneration in a critical sized tibial defect model [142].

**Figure 2:**
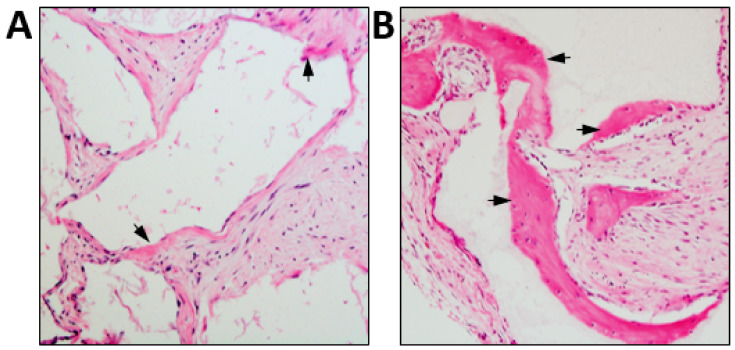
Differential osteoconductive properties of bio-materials. Culture expanded human BMSC were seeded onto (**A**) JAX 100% β-TCP (Smith & Nephew) or (**B**) Master Graft 15% HA/80% β-TCP granules (Medtronic Sofamor Danek), then transplanted subcutaneously into immunocompromised NOD-SCID mice for eight weeks. Representative cross sections of each transplant are shown stained with H&E depicting new bone formation (arrows). Magnification 200x.

The use of HA composites seeded with autologous BMSC to repair non-union fractures of long bones was first reported by Quarto and colleagues [143]. In 2010, the Australian Therapeutic Goods Administration (TGA) issued the world’s first license to Mesoblast Ltd. to supply ex vivo expanded autologous mesenchymal precursor cells for use in the repair and regeneration of long bone fractures after trauma, stress fractures following sporting injury, and vertebral fractures due to osteoporosis. The license was granted following a phase 1 clinical safety trial utilizing 100–200 × 10^6^ autologous cells seeded onto HA/TCP collagen scaffolds per patient, which resulted in the fusion of non-union fractions in 9 out of 10 patients. Currently there is a multicenter (ORTHOUNION) open-label, three-arm, randomized comparative phase 3 clinical trial (EudraCT number 2015-000431-32) in progress. This trial is investigating skeletal repair of patients that have experienced long bone (femur, tibia or humerus) diaphysis and/or metaphyso-diaphyseal fractures where the patient status was atrophic or oligophic nonunion for greater than 9 months. The trial is initially addressing whether a low (100 × 10^6^ cells) or a high (200 × 10^6^ cells) dose of autologous human BMSC, in combination with human albumin and the granulated biomaterial MBCP+ improves skeletal repair when compared to the standard autograft from the iliac crest [35]. However, at present there is no available data relating to this study outcomes. In other trials, autologous MSC therapy have been used to treat non-union fractures in diabetic patients [144]. Autologous MSC treatment displayed favorable outcomes when compared to standard care bone-graft treatment of non-union fractures in diabetic patients [145]. Furthermore, while BMSC from diabetic and non-diabetic patients display similar proliferative capacity as demonstrated by colony forming unit-fibroblast (CFU-F) assays, the capacity for the autologous BMSC to facilitate frequency of union, time of healing and callus volume were impeded in the diabetic patients. In diabetic patients, bone synthesis and quality/fracture healing is impeded due to the dysregulation in molecular signals influencing adipogenic, osteogenic and osteoclastic formation and function [146]. Therefore, it has been proposed that diabetic patients should be administered a higher concentration of BMSC than non-diabetics to achieve better outcomes [144]. It has also been suggested that stimulating BMSC response to IGF-1 signaling may improve fracture healing as demonstrated in a rodent preclinical model [145,147]. Therefore BMSC dosing, choice of biomaterial (Figure 2) and addition of exogenous factors may help optimize the efficacy of BMSC based therapy for skeletal regeneration and repair.

### 3.2. Bone Related Disease—Osteogenesis Imperfecta

Osteogenesis Imperfecta (OI) is a congenital disorder caused by different dominant and recessive mutations in the collagen genes, *COL1A1* and *COL1A2* result in OI type I-IV. However, causative genes whose proteins normally associate with collagen also result in rare recessive forms of OI, including types V-XII and unclassified as reviewed by Marini and colleagues [148]. Depending on the severity, the pathophysiology predominantly results in reduced collagen quantity or abnormal collagen microfibril assembly, while the other causative genes influence osteoblast development, matrix mineralization and hydroxylation defects [91,148]. The most common clinical manifestations include abnormal skeletal development, short stature, and skeletal fragility among other non-skeletal related indications [93,149,150]. Preclinical OI mouse studies have reported that MSC transplantation may be a viable option to treat OI [51,91,149,151,152]. Furthermore, priming blood-derived human fetal MSC with CXCL12 to upregulate CXCR4 expression enriches donor cell engraftment, improves bone mechanisms and reduces bone brittleness in a mouse OI model [150]. Work conducted by Horwitz and colleagues was the first to demonstrate the utility of MSC therapy to treat OI patients. The initial clinical studies demonstrated safety and efficacy of intravenous infusion of HLA-matched allogeneic unmanipulated bone marrow in children with OI. Patient outcomes improved with increased growth and decreased incidences in bone fractures observed over a 36 month period [153,154]. This was followed by clinical studies investigating the efficacy of administering two courses of culture expanded allogeneic MSC [155] due to the limiting influence of MSC administration. In one case study fetal implantation of MSC to a 32-week old fetus with OI was assessed as a potential treatment option [156]. Other studies have demonstrated that prenatal transplantation of allogeneic fetal liver derived-MSC are safe and beneficial, and that subsequent transplantation with the same fetal liver-derived MSC at 8 years of age was feasible. It is worth noting that the number of patients in this study was low and therefore requires further ivalidation with increased patient numbers [157]. A European clinical trial (NCT03706482) known as the “Boost Brittle Bones Before Birth (BOOSTB4)” which is utilizing liver-derived MSC is currently recruiting patientas. Whilst another phase 1 clinical trial (NCT02172885) based on MSC infusion has treated two patients, there are no publications relating to the outcomes of this study. Furthermore, Horwitz and colleagues have demonstrated that intravenous infusion of T-cell depleted BMMNC, comprising <0.01% MSC to patients (NCT00187018) that had previously received bone marrow transplant resulted in cellular engraftment and accelerated growth in some patients [51]. 

Understanding the cellular and molecular mechanisms contributing to stem cell derived skeletal repair in OI is of particular interest. It has been proposed that BMMNC contain osteoprogenitors that are able to form normal collagen following their differentiation into osteoblasts [51]. While, MSC predominantly mediate a paracrine response indirectly stimulating chondrogenic activity with the growth plate [51]. Therefore an alternative approach has been postulated to treat OI. This approach utilizes a cell-free strategy of infusing extracellular vesicles secreted by MSC [158]. This approach showed some promising outcomes in a mouse model of OI with improved bone growth that was mediated by stimulating chondrogenesis within the growth plate [158]. Fisk and colleagues also noted a lack of donor-derived osteogenesis within the bone following intrauterine bone marrow transplant in an OI mouse model. However, osteoclasts and osteomacs derived from the bone marrow transplant were detected within the epiphyseal and metaphyseal regions [159]. This observation is also supported by lineage tracing studies demonstrating hematopoietic lineage engraftment following bone marrow transplantation [160]. Collectively these findings suggest that MSC treatment for OI appears to be of some benefit to the patient, however further research is required to further elucidate the mechanism by which MSC therapy or combined bone marrow transplantation improve skeletal outcomes in individuals with OI. 

Genetic manipulation of MSC is another promising approach to treat OI, which was first explored in the early 2000s [161,162,163,164]. More recently human derived induced pluripotent stem cells (hiPSC) from OI patients or engineered iPSC to replicate OI have been generated. In combination with in vitro assays and in vivo models, these iPSC are being used to investigate therapeutic genome editing strategies and to test drug targets which may rescue the OI phenotype [165,166,167]. Notably, a proof-of-concept study demonstrated that MSC derived iPSC from OI patients, termed iMSC, could be genetically manipulated with adeno-associated virus mediated gene targeting to correct the mutant collagen genes. These genetically modified iMSC produced bone when seeded onto HA/TCP and implanted into immunocompromised mice [165]. While these finding are encouraging, further verification is needed to demonstrate efficacy and safety of these approaches using preclinical OI models. 

### 3.3. Repair of Craniofacial Bone

Cranial defects are a global healthcare burden, where defects can arise from dental caries, periodontal disease, oral cancer, osteonecrosis of the jaw during treatment of osteoporosis, in addition to mandibular defects and congenital defects such as craniofrontonasal syndrome (CFNS), cleft palate and cleft lip. Traditional treatments have utilized autologous grafts originating from the iliac crest, fibula, ribs or scapula. While these approaches are somewhat effective, they are coupled with secondary morbidity sites and restricted access to these tissues. 

Recently it has been proposed that a cell-free approach utilizing small molecules such as CXCL12, or exosome mimetics isolated from hMSC may be worthwhile pursuing clinical [168,169,170]. This approach was examined using a rodent preclinical calvarial defect model [170]. Alternatively conditioned media derived from hMSC under cyclic stretch conditions may also be advantageous in stimulating cranial bone repair and vascular infiltration through the secretion of paracrine factors that promote osteogenesis and angiogenesis [171]. The use of endogenous and exogenous MSC have also been utilized to treat critical sized defects within the cranium with some success. A proof-of-concept study demonstrated that a rapidly resorbable scaffold consisting of calcium sulfate/simvastatin-controlled microspheres augmented calvarial bone synthesis in a rat cranial critical sized defect model. This scaffold was osteo-conductive and osteo-inductive, with enriched osteogenic and angiogenic activity [172]. Building on this concept a coral derived HA-collagen hybrid scaffold called Coll/Pro Osteon 200 has also been developed which promoted bone regrowth in patients that were treated for maxilla-mandibular malocclusion and/or asymmetry. A 36-month follow-up demonstrated that this scaffold was efficacious, improving bone growth, where the scaffold had been resorbed over time and replaced with cortical bone [173]. While these studies did not directly investigate the infiltration of endogenous MSC, it could be presumed that endogenous MSC were recruited based on the enrichment in bone formation. 

Alternative tissue engineering approaches to treat critical size defects within the cranium or to access exogenous MSC, have been coupled with osteo-conductive/biocompatible biomaterial scaffolds; or with inductive factors or alternative cellular populations, which have the capability to mimic the bone environment and potentiate bone regeneration [174,175,176,177]. Certainly this later approach has shown some promise in rodent proof-of-concept preclinical cranial critical size defect models [178,179]. Where allogeneic BMSC pre-seeded onto specialized bio-compatible scaffolds containing BMPs, particularly BMP2, BMP6 and BMP9, augmented bone regeneration [178,180]. Furthermore, it was also established that CXCL12 in combination with BMP further enhanced bone repair [179]. While this strategy has not yet been implemented in the clinic, several clinical studies have been conducted utilizing exogenous stem cells although few have investigated BMSC stem cell based treatment options. Rather dental derived cell options have been employed for dental, periodontal, gingival and bone regeneration clinical trials, including dental pulp, gingival and periodontal ligament derived stem/progenitor cells [181,182,183,184,185,186,187,188,189]. 

The first randomized controlled clinical trial (ClinicalTrials.gov number CT00755911) for the treatment of localized jaw bone defect used culture expanded autologous bone marrow derived cells placed within Gelfoam, an absorbable gelatin sponge. This trial showed that treatment with the bone marrow population was efficacious, feasible and safe when compared to the conventional guided bone regeneration procedure [50]. Although these cells were not BMSC as such, the population did include CD90+ cells, in addition to CD14+ monocyte/macrophage population. Collectively, this population resulted in greater tissue regeneration that appeared to also display greater vascularity and implant stability that required less secondary bone grafting than the control group [50]. The therapeutic potential of this population of bone marrow derived CD90+ cells and CD14+ monocytes, this time combined with a β-TCP scaffold, also improved maxillary sinus bone regeneration in a randomized clinical trial (Clinicaltrials.gov NCT00980278). The engineered alveolar bone showed greater bone density and quality, where no adverse events were recorded 1-year follow-up to implantation of the functionally loaded dental restoration [49]. More recently a pilot study utilizing autologous BMSC grafts to treat medication-related osteonecrosis of the jaws also healed appropriately [190]. Collectively these studies suggest that tissue engineered MSC therapy for the treatment of cranial bone defects is beneficial.

Periodontitis is a chronic multifactorial inflammatory disease caused by exposure to Gram-negative bacteria including *P gingivalis* and *F nucleaturm*. This exposure leads to bone loss, periodontal ligament destruction and tooth loss. This is mediated through the enhanced recruitment of monocytes and macrophages in addition to inflammatory cells which negatively influence the bone remodeling process through elevated Receptor Activator of Nuclear Factor Kappa B Ligand (RANKL) and Transforming Growth Factor Alpha (TNFα) signaling and thus increased osteoclast activity. There are limited clinically available therapeutic treatments for periodontitis apart from mechanical or surgical debridement with grafting procedures. Therefore stem cell based approaches have been explored to improve functional regeneration of lost bone [182]. In a phase I/II clinical trial based on preclinical studies, autologous BMSC combined with platelet-rich plasma, and a biodegradable 3D woven-fabric composite scaffold showed efficacy, stability and safety for periodontists over a 36 month period [54]. Importantly the platelet rich plasma consists of various growth factors essential for proliferation and differentiation [191]. Collectively these studies along with a number of others described in this review suggest that stem cell treatment combined with either a heterogeneous population of cells or plasma appears to be efficacious for bone regeneration by recapitulating to some degree the in vivo bone microenvironment.

## 4. Conclusions/Summary

Whilst the use of tissue engineering for skeletal repair is a complex undertaking, it has been shown to be a feasible approach for mediating bone regeneration, through the exploitation of the multi-faceted characteristics of MSC. Over the last decade, significant advancements have been made in the field of bone tissue engineering through interdisciplinary collaborations. These advances have led to the generation of novel hydrogels and biomimetic scaffolds as cell-free delivery systems, and the use of MSC alone, their products or in combination with biomaterials and/or bioactive molecules to attain the appropriate mechanical, cellular and regenerative properties required to recapitulate bone structures. This work has made considerable headway into the clinic, with encouraging outcomes being reported for non-union fracture repair. However, further studies are still required to build on current preclinical and clinical studies in order to address limitations in facilitating tissue and site specific osseous repair. In particular, more detailed assessment is required to understand the heterogeneity of different stromal populations and their products or factors that contribute to bone synthesis. Importantly, the role of resident cell populations within the bone microenvironment, require further investigation to identify the mechanisms driving bone regeneration. It is anticipated that future advances in MSC based therapies would also benefit from the inclusion of adjuvant strategies (such as plasma products) and the manipulation of other cellular components (such as monocytes, pre-osteoclasts and endothelial cells), which help recapitulate and maintain the bone microenvironment. This could be facilitated through the use of scaffold based systems to deliver small molecules and/or genetic modified BMSC for more directed and controlled skeletal tissue regeneration. 

## Figures and Tables

**Table 1:** Clinical Trials in Fracture Repair. Search criteria in ClinicalTrails.gov: BMSC and bone fracture, scaffold and bone fracture, stem cells and scaffold and bone fracture. Terminated, suspended and withdrawn trials and cartilage related trials have been removed from the table.

Year First Posted/Updated Clinical Trial Number	Brief Title	Status	Intervention	Trial Type	Citation
2017/NCT03325504 EudraCT number 2015-000431-32	A Comparative Study of 2 Doses of BM Autologous H-MSC + Biomaterial vs Iliac Crest AutoGraft for Bone Healing in Non-Union (ORTHOUNION)	Ongoing	Autologous BMSC + granulated biomaterial MBCP+	Open-Label, Randomized, Comparative Clinical Trial	[35]
2019/2020 NCT03884790	Pre-market Study to Evaluate Safety and Performance of GreenBone Implant (Long Bone Study)	Ongoing	Implantation of new generation bone graft “GreenBone” within the long bone defect	Open-labeled clinical trial	
2016/2020 NCT02803177 EudraCT number 2015-001820-51	Cell Therapy by Autologous BMC for Large Bone Defect Repair (BMC2012)	Completed	Implantation of BMMNC seeded onto β-TCP and stable fixation	Single blind, randomized, Phase II trial	
2015/2020 NCT02566655 EudraCT number 2012-005814-20	Clinical Trial of Intravenous Infusion of Fucosylated Bone Marrow Mesenchyme Cells in Patients With Osteoporosis (CSM/OP/2011)	Complete	Intravenous injection fucosylated BMSC	Open-labelled Phase II trial	
2014/2020 NCT02177565	Autologous Stem Cell Therapy for Fracture Non-union Healing	Complete	Carrier combined with in vitro expanded autologous BMSC	Randomized, double blind Clinical trial	
2007/2018 NCT00512434	Percutaneous Autologous Bone-marrow Grafting for Open Tibial Shaft Fracture (IMOCA)	Completed	Percutaneous injection of autologous BMMNC	Open-label, randomized, clinical trial	
2013/2017 NCT01842477 EudraCT number 2011-005441-13	Evaluation of Efficacy and Safety of Autologous MSCs Combined to Biomaterials to Enhance Bone Healing (OrthoCT1)	Completed	Implantation of cultured autologous BMSC coupled with granulated biphasic calcium phosphate	Open-label, Phase I/II trial	[36]
2013/2017 NCT01813188	Clinical Trial Based on the Use of Mononuclear Cells From Autologous Bone Marrow in Patients With Pseudoarthrosis	Completed	Transplantation of autologous BMMNC combined with porous TCP and DBM	Open-label, randomized, Phase II trial	
2016/2016 NCT02910232	In Vivo Clinical Trial of Porous Starch - Hydroxyapatite Composite Biomaterials for Bone Regeneration	Completed	Implantation of “bone void filler” comprised of porous starch HA composite	Observations clinical trial	
2014/2016 NCT02153372	Cell Therapy by Bone Marrow-derived Mononuclear Cells (BMC) for Large Bone Defect Repair: Phase-I Clinical Trial (BMC2012)	Completed	Implantation of BMMNC seeded onto β-TCP and stable fixation	Open-label, Phase I trial	[37]
2015/2015 NCT02448849	Autologous BM-MSC Transplantation in Combination With Platelet Lysate (PL) for Nonunion Treatment	Unknown	Precutanteous injection of autologous BMSC combined with platelet lysate	Randomized, double blind Phase II/III	
2012/2015 NCT01581892	Use of Adult Bone Marrow Mononuclear Cells in Patients With Long Bone Nonunion	Completed	Infusion of autologous BMMNC with an “osteogenic matrix” within the fracture site	Open-label, Non-randomized, Phase I/II trial	[38]
2015/2015 NCT02609074	Pilot Clinical Trial of CPC/rhBMP-2 Microffolds as Bone Substitute for Bone Regeneration	Completed	Implantation of CPC/rhBMP-2 micro-scaffold	Open-label, randomized, Phase IV trial	[39]
2014/2014 NCT02307435	Allogenic Mesenchymal Stem Cell for Bone Defect or Non Union Fracture (AMSC)	Unknown	Implantation of allogenic MSC derived from either umbilical cord, bone marrow or adipose directly or following cryopreservation.	Open label - Phase I trial	
2013/2013 NCT01788059	The Efficacy of Mesenchymal Stem Cells for Stimulate the Union in Treatment of Non-united Tibial and Femoral Fractures in Shahid Kamyab Hospital	Completed	Injection of BMSC	Open-labelled Phase II trial	
2013/2013 NCT01958502	Evaluation the Treatment of Nonunion of Long Bone Fracture of Lower Extremities (Femur and Tibia) Using Mononuclear Stem Cells From the Iliac Wing Within a 3-D Tissue Engineered Scaffold	Unknown	Transplant of MSC with BMP2 in a collagen scaffold	Open-labelled Phase II trial	
2011/2013 NCT01429012	Treatment of Atrophic Nonunion Fractures by Autologous Mesenchymal Stem Cell Percutaneous Grafting	Unknown	Injection of autologous BMSC	Randomized, quadruple blind Phase II/III trial	
2009/2012 NCT00916981	Treatment of Atrophic Nonunion by Preosteoblast Cells	Completed	Percutaneous implantation of autologous cultured preosteoblasts	Open-label, Phase I/II trial	
2010/2011 NCT01206179	Treatment of Non Union of Long Bone Fractures by Autologous Mesenchymal Stem Cell	Complete	Injection of BMSC with platelet lysate	Open-label Phase I trial	[40,41]
2005/2011 NCT00250302	Autologous Implantation of Mesenchymal Stem Cells for the Treatment of Distal Tibial Fractures	Complete	Injection of autologous BMSC with PRP and DBM within the fracture site.	Prospective randomized, open-label controlled Phase I trial.—27 patients	[42]
2011/2014 NCT01435434	Mononucleotide Autologous Stem Cells and Demineralized Bone Matrix in the Treatment of Non Union/Delayed Fractures	Unknown	Injection of autologous bone marrow cells in conjunction with ^®^ICS injectable scaffold	Open-label, clinical trial	

Legend: BM—Bone marrow, BMSC—Bone marrow stem cells, BMMNC—bone marrow mononuclear cells, MSC—mesenchymal stem cells, PRP—plasma rich plasma, DBM—demineralized bone matrix, CPC—calcium phosphate cement, rhBMP—recombinant human bone morphogenetic protein, BMP2— bone morphogenetic protein 2, HA—hydroxyapatite, β-TCP—beta tri-calcium phosphate.

**Table 2:** Clinical Trials in Bone Regeneration. Search criteria in ClinicalTrails.gov: BMSC and bone regeneration, scaffold and bone regeneration, stem cells and scaffold and bone regeneration. Terminated trials, fracture related trials and cartilage related trials removed from the table.

Year First Posted/Updated Clinical Trial Number	Brief Title	Status	Intervention	Trial Type	Citation
2020/2020 NCT04297813 EudraCT, 2018-001227-39	Efficacy in Alveolar Bone Regeneration With Autologous MSCs and Biomaterial in Comparison to Autologous Bone Grafting (Maxibone)	Ongoing	Augmentation of alveolar ridge with culture expanded autologous MSC coupled with biphasic calcium phosphate	Open-label, randomized, Phase III trial	
2018/2020 NCT03417375	Assessment of the Quality and Quantity of Bone Regeneration	Completed	Augmentation of maxillary sinus with the “Osteocel Plus” graft	Single blinded, randomized clinical trial	[43]
2016/2020 NCT02751125 EudraCT, 2012-003139-50	Reconstruction of Jaw Bone Using Mesenchymal Stem Cells	Completed	Augmentation of narrow alveolar ridge with cultured autologous BMSC combined with bi calcium phosphate	Open-label, Phase I trial	[44,45]
2013/2020 NCT01878084	Bioactive Glass (Sol-gel) for Alveolar Bone Regeneration After Surgical Extraction	Completed	Implantation of bioactive glass scaffold (Sol-gel) within the alveolar bone following extracted mandibular and maxillary premolars	Open-label, Phase I/II trial	[46]
2018/2019 NCT03706482 EudraCT, 2015-003699-60	Boost Brittle Bones Before Birth (BOOSTB4)	Ongoing	Osteogenesis Imperfecta–Multi dose (x4) intravenous administration allogenic expanded fetal-MSC	Open-label, non-randomized, Phase I/II trial	
2012/2017 NCT01603693	Bone Quality and Quantity Following Guided Bone Regeneration Prior to Dental Implant Placement	Completed	Assessment of guided bone regeneration procedure comparing the use of a DBBM “Bio-Oss” alone or coupled with bi-phasic calcium sulphate (BONDBONE)	Open-label, randomized, clinical trial	
2011/2017 NCT01389661	Treatment Of Maxillary Bone Cysts With Autologous Bone Mesenchymal Stem Cells (MSV-H) (BIOMAX)	Complete	Transplantation of cultured autologous BMSC with autologous plasma matrix serum cross-linked scaffold (BioMAx) into cavity of removed cyst in the maxillofacial region	Open-label, Phase I/II trial	[47]
2011/2017 NCT01361321	Bone Quality and Quantity Following Guided Bone Regeneration	Completed	Assessment of guided bone regeneration procedure	Observational study	
1999/2017 NCT00001391	Bone Regeneration Using Bone Marrow Stromal Cells	Completed	No details provided	Observational study	
2015/2016 NCT02396056	Enhancing Guided Bone Regeneration by Modifying a Resorbable Membrane	Unknown	Augmentation of alveolar ridge with modified bovine perforated collagen membrane (MPM)	Single blind, randomized clinical trial	
2011/2016 NCT01323894	Osteogenic Effects in Human Mesenchymal Stem Cells Enhanced by Wnt Signaling	Completed	Comparison between non-viral and viral administration of Wnt3a human MSC with HA nanoparticles	Observational study	
2014/2017 NCT02293031	Gene-activated Matrix for Bone Tissue Repair in Maxillofacial Surgery	Unknown	Implantation of gene-activated matric “Nucleostem” within maxillofacial bone defects or alveolar bone atrophy	Open-labeled clinical trial	[48]
2015/2015 NCT02523651	Periodontal Regeneration of Chronic Periodontal Disease Patients Receiving Stem Cells Injection Therapy	Unknown	Local injection of allogenic DPSC into affected periodontal tissue	Triple blind, randomized, Phase I/II trial	
2015/2015 NCT02639572	Evaluation of SilOss^®^ in Periodontal Surgery	Completed	Implantation of Siloss^®^ bone graft (composed of dicalcium phosphate anhydrous, HA, amorphous silica and trace amounts of zinc) within intrabony defect	Double-blind, randomized, Phase II trial	
2009/2015 NCT00980278	Bone Tissue Engineering Using Autologous Bone Repair Cell (BRC) Therapy for Sinus Floor Bone Augmentation	Completed	CD90+ autologous Stem cells and CD14+ monocytes with beta-tri calcium phosphate scaffold	Randomized, Phase I/II trial	[49]
2008/2015 NCT00755911	Treatment of Alveolar Bone Defects Using Aastrom Biosciences Autologous Tissue Repair Cell Therapy	Completed	Bone Marrow derived CD90+ CD14+ stem cells with absorbable gelatin sponge	Open-label, randomized, Phase I/II, controlled feasibility trial	[50]
2005/2015 NCT00187018	Marrow Mesenchymal Cell Therapy for Osteogenesis Imperfecta: A Pilot Study	Completed	Allogeneic bone marrow transplant following removal of CD3+ cells	Open-label, clinical trial	[51]
2010/2010 NCT01105026	A Clinical Investigation to Evaluate the Healing of Tooth Extraction Sites Filled With BioRestore^TM^	Completed	Implantation of bioactive glass scaffold (BioRestore^TM^) within freshly extracted tooth socket/alveolar bone	Open-label, Phase I trial	[52,53]
2009/2009 NCT00836797	Radiographic Assessment of Bone Regeneration in Alveolar Sockets With PLGA Scaffold	Completed	Administration of PLGA bioscaffold following tooth extraction	Case-control, Phase I trial	
2005/2009 NCT00221130	Clinical Trials of Regeneration for Periodontal Tissue	Completed	Periodontitis-Autologous BMSCs-PRP/3D woven-fabric composite scaffold	Open-label, non-randomized, Phase I/II trial	[54]

Legend: BMSC—Bone marrow stem cells, MSC—mesenchymal stem cells, DPSC—dental pulp stem cells, PLGA—Poly Lactic-co-Glycolic Acid, PRP—plasma rich plasma, DBBM—deproteinized bovine bone mineral, HA—hydroxyapatite.

## Abbreviations

3D	Three dimensional
α	Alpha
β	Beta
BMMNC	Bone marrow mononuclear cells
BMP	Bone morphogenetic protein
BMSC	Bone marrow stromal/stem cells
Ca	Calcium
CA	Catechol
CA-CS/ZM	Ca/cs hydrogel modified by zif-8 np at a medium (1.2 mg) composition
CAG	Chitosan-agarose-gelatin
CaP	Calcium phosphate
CD	Cluster of differentiation
CFNS	Craniofrontonasal syndrome
CFU-F	Colony forming unit-fibroblast
CPC	Calcium phosphate cement
CS	Chitosan
CSO/H	Chitosan oligosaccharide/heparin
Cu	Copper
CXCL12	C-X-C Motif Chemokine Ligand 12
CXCR4	C-X-C Chemokine Receptor type 4
DBBM	Deproteinized bovine bone mineral
DBM	Demineralized bone matrix
DPSC	Dental Pulp Stem Cells
ECM	Extracellular matrix
EGF-R	Epidermal Growth Factor Receptor
ELP	elastin-like proteins
FDA	Food and Drug Administration
HA	Hydroxyapatite
hiPSC	human derived induced pluripotent stem cells
HLA-DR	Human Leukocytet Antigen-DR
hMSC	Human mesenchymal stem cells
IGFBP5	Insulin-like Growth Factor Binding Protein 5
IGF-R	Insulin-like Growth Factor Receptor
IL-10	Interleukin 10
K	Potassium
Lrg5	Leucine Rich Repeat Containing G Protein-Coupled Receptor 5
Mg	Magnesium
NGF-R	Nerve Growth Factor Receptor
OI	Osteogenesis Imperfecta
P	Phosphorus
PAM	Polyacrylamide
PDGF-R	Platelet-Derived Growth Factor Receptor
PEG	Poly(ethylene glycol)
PLGA	Poly Lactic-co-Glycolic Acid
PRP	Plasma rich plasma
PTH	Parathyroid Hormone
PTH1R	Parathyroid Hormone 1 Receptor
PVA	Plyvinyl alcohol
RANKL	Receptor Activator of Nuclear Factor-Kappa B
SiO_2_	Silicon dioxide
TCP	Tri-calcium phosphate
TGA	Therapeutic Goods Administration
Ti	Titanium
TiO_2_	Titanium dioxide
TNFα	Tumor Necrosis Factor alpha
VEGF	Vascular Endothelial Growth Factor
ZA	Zoledronic acid
ZIF-8 NP	Zeolitic imidazolate framework-8 nanoparticle
Zn	Zinc
ZnO	Zinc oxide
ZrO_2_	Zirconium oxide
